# A Web-Based Intervention for Young Adults Whose Parents Have a Mental Illness or Substance Use Concern: Protocol for a Randomized Controlled Trial

**DOI:** 10.2196/15626

**Published:** 2020-06-18

**Authors:** Darryl Maybery, Andrea Reupert, Catherine Bartholomew, Rose Cuff, Zoe Duncan, Kim Foster, Jodie Matar, Laura Pettenuzzo

**Affiliations:** 1 School of Rural Health Monash University Warragul Australia; 2 Faculty of Education Monash University Clayton Australia; 3 Wellways Melbourne Australia; 4 Bouverie Centre Melbourne Australia; 5 School of Nursing, Midwifery & Paramedicine Australian Catholic University Melbourne Australia; 6 North Western Mental Health, Melbourne Health Melbourne Australia

**Keywords:** young adult, mental health, substance use, internet-based intervention

## Abstract

**Background:**

One in 5 young people grow up in a family where one parent has experienced a mental health problem or substance use concern. Compared with their same-aged peers, these youth are at a higher risk of academic failure and acquiring a substance abuse and/or mental health issue. There is a paucity of accessible, age-appropriate interventions that address their needs.

**Objective:**

A 6-week, web-based intervention, “mental illness: supported, preventative, online, targeted” (mi.spot), was developed based on previous research and the competence enhancement model. This paper describes the protocol for a randomized controlled trial and details how the usage, safety, acceptability, and feasibility of the intervention will be determined.

**Methods:**

Participants will be recruited through social media and clinician referral. A total of 70 Australians, aged 18 to 25 years, who grew up with parents with a mental illness or substance use concern will participate in a 2-arm parallel randomized controlled trial. The assessment will consist of a baseline measurement and 2 follow-up periods, posttest and 6-week follow-up, using the Mental Health Continuum short form; the Depression, Anxiety, and Stress Scale; the Coping Orientation to Problems Experienced inventory; the General Help Seeking Questionnaire; the Social Connectedness Scale; the Mental Health Literacy Scale; the General Self-Efficacy Scale; and the Attribution of Responsibility for Parental Mental Illness Measure. Impact will be examined at pre, post, and follow-up time periods using analyses of variance that will include a within-subjects factor (time) and a between-subjects factor (intervention/control). Facilitator interviews will ascertain intervention feasibility. Participant interviews will ascertain intervention acceptability. Interview data will be analyzed within a qualitative framework. Usage (data analytics) across site features and several indicators of clinical safety will also be reported.

**Results:**

The impact of mi.spot will be examined at pre, post, and follow-up time periods using analyses of variance on each of the measures outlined above. There will be a within-subjects factor (time) and a between-subjects factor (intervention/control). Data analysis will employ the intention-to-treat principle by including all participants in the analyses. Qualitative interview data will be analyzed using interpretative phenomenological analysis along with respondent validation. The Monash University Human Research Ethics Committee (reference number: 2019-18660-30434) approved the trial on April 17, 2019. As of October 2, 2019, 30 participants were enrolled in the control group and 34 participants were enrolled in the intervention group. Result are expected to be submitted for publication in December 2020.

**Conclusions:**

Study results will provide reliable evidence on a web-based intervention that has the potential to make a difference to the lives of many vulnerable young adults. Implementation guidelines are needed to embed the intervention in different service sectors.

**Trial Registration:**

Australian New Zealand Clinical Trials Registry ACTRN12619000335190; https://anzctr.org.au/Trial/Registration/TrialReview.aspx?ACTRN=12619000335190

**International Registered Report Identifier (IRRID):**

DERR1-10.2196/15626

## Introduction

### Background

A systematic review recently substantiated that up to 45% of clients in adult mental health services are parents with children [1]. Maybery et al [2] estimated that more than 1 in 5 children have 1 parent with a mental health problem. Compared with their same-aged peers, these children are at high risk of school dropout and failure [3], being taken into care [4], and acquiring a substance misuse concern and/or mental illness [5]. Their problems often continue into adulthood; a 30-year follow-up found that the risk of major depression was approximately three times as high in the children whose parents had depression, with the period of highest risk for first onset between 15 and 25 years of age [6]. Given the prevalence and needs of this at-risk group of young people, it is imperative that there are evidence-based, easily accessible interventions targeted at their specific needs.

One of the traditional ways of supporting young people in these families has been through face-to-face peer support groups [7]. These aim to prevent the onset of mental health problems in young people by providing social support, psychoeducation, and training in adaptive coping [7]. In a randomized controlled trial based in the Netherlands, van Santvoort et al [8] showed that children in such an intervention experienced a greater decrease in negative cognitions and sought more social support compared with the control group. Notwithstanding these benefits, face to face programs have several recruitment issues related to stigma, referral pathways, transport, and time [9]. There are also relatively fewer programs for young people living in rural and remote areas compared with their urban counterparts [10]. In addition, face-to-face peer support programs target children aged 12 to 18 years and not older youth aged 18 to 25 years [7].

### Web-Based Interventions

Web-based interventions have the potential to circumvent stigma, reach, and access issues. Furthermore, young adults are increasingly turning to the internet to search for health information and to share personal information [11,12] because it is highly engaging, accessible, anonymous, and often free of charge [13]. Young adults living in these families have indicated a preference for online support [14] with specific preferences for topics on psychoeducation, managing the parent-child relationship, and strategies to build resilience and improve coping and mental health [15]. There are some online interventions for youth aged 18 to 25 years whose parents have a mental illness/substance use concern, although none are in English and are still in the early stages of development [16-18]. For example, a randomized controlled trial in the Netherlands, of an online intervention called Kopstoring, found positive trends toward a reduction in internalizing symptoms but no significant differences in self-reported depressive symptoms and internalizing problems [18]. Further work is needed to consolidate and substantiate the evidence base in this area and ensure that interventions designed for this group are effective.

### Objectives

This paper describes the protocol for a randomized controlled trial for the mental illness; supportive, preventative, online, targeted (mi.spot) intervention. It is hypothesized that following the mi.spot intervention, young adults will report the following:

- Significant improvements in mental health and well-being (primary outcomes)
- Significant improvements in coping, social connectedness, and attribution of responsibility for parental mental illness (secondary outcomes)
- Significant increases in help seeking and mental health literacy (secondary outcomes).

Furthermore, the study will determine what components of the site participants use and do not use, along with their views about intervention safety and acceptability. Facilitators’ views of the feasibility of the intervention will also be sought.

## Methods

### Design

This 2-arm parallel randomized controlled trial will compare outcomes at posttest and 6-week follow-up for mi.spot and control participants. The protocol is in accordance with the Consolidated Standards of Reporting Trials of Electronic and Mobile HEalth Applications and onLine TeleHealth checklist. The study was undertaken in 2019. The protocol was registered by the Australian and New Zealand Clinical Trials Register on May 5, 2019. The registration number is ACTRN12619000335190.

### Ethics

For inclusion, all participants must give implied consent online before completing the questionnaires. Written consent is required for the interviews. Ethics approval for this study was obtained from the Monash University Human Research Ethics Committee (reference number: 2019-18660-30434) on April 17, 2019.

### The Intervention

**Figure 1:**
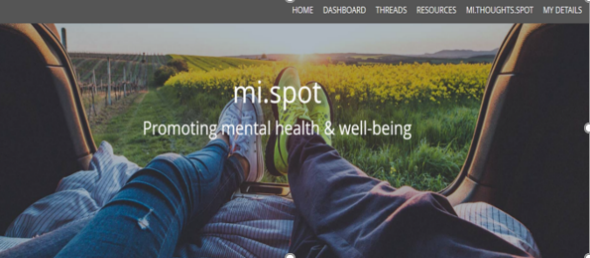
Screenshot of the mental illness; supportive, preventative, online, targeted intervention.

mi.spot is a 6-week professionally moderated online intervention for emerging adults (aged 18-25 years inclusive) who have a parent with a mental illness or substance use concern (Figure 1). It is based on the competence enhancement model, which incorporates cognitive behavioral principles and a strengths-based approach [19]. A reference group was formed to guide intervention development, consisting of expert clinicians, researchers, and young adults with lived experience. Drawing on the known risk and resilience factors for this particular group of young adults [20], the intervention aims to improve psychoeducation; increase adaptive coping, connectedness, and knowledge about healthy relationships; encourage help-seeking behaviors; decrease feelings of attribution about their parent’s illness; and foster well-being and mental health (refer to the paper by Reupert et al [21] for further details regarding the theoretical background and empirical rationale for the intervention).

The site is anonymous, and participants give themselves a nickname they use in all online interactions. All features are optional, and participants may choose to *lurk* rather than actively contribute. The approach includes 6 professionally facilitated online weekly chats that run for 1 hour a week on set topics with accompanying video, audio, and print resources (Table 1). The accompanying resources are made available when the accompanying session is offered. There are also opportunities for private one-to-one online counseling sessions between the participant and the facilitator, which can be initiated by either the participant or by one of the facilitators if they believe the participant requires additional support.

The site includes mi.thoughts.spot, which functions as an asynchronous, online private diary for participants to use and which is visible only to the individual participant and facilitator. mi.thoughts.spot allows participants to record their feelings, practice reframing automatic negative thoughts and challenge unhelpful beliefs using a cognitive behavioral approach. There are also opportunities for participants to chat with each other on group threads. The facilitator’s role is to monitor the site, encourage young adults to apply a strength-based cognitive behavioral model, facilitate weekly topics, and promote healthy peer sharing and support.

**Table 1:** The weekly mental illness; supportive, preventative, online, targeted topics.

Topic	Description
What is mi.spot all about?	This session introduces the site and provides participants with an opportunity to get to know each other and the facilitators. Guidelines (rules) for using the site are described, and the various components of the site are described. The cognitive behavioral approach is explained and practiced.
Learning more about mental health and illness	This session delivers basic psychoeducation about different types of mental illnesses. Participants are invited to reflect on what they know about their parent’s illness or substance use issue, what they want to know, and how their parents’ illness might impact their mental health and well-being. Specific genetic vulnerabilities are discussed. Ways to promote well-being are emphasized.
Me, my parent, and other relationships	In this session, participants reflect on their relationship with their parent(s) and consider how these relationships might inform other intimate relationships. Strategies for forming healthy relationships and boundary setting are also shared.
Managing stress	In this session, participants identify a current stressor and consider what they did or might do to manage this. They are prompted to share adaptive coping strategies and useful ways to regulate emotions.
Caring—who me?	Participants are invited to describe any caring responsibilities they may have and the potential positive and negative impacts that caring for others might have. The principles of self-care and self-compassion are also covered.
Taking control of my life	In the final week, participants consider what they have learnt over the 6 weeks. This session also covers help-seeking strategies, including an emphasis on asking for help early, who they might turn to, and how they might ask for help. A list of relevant services is shared.

### Setting and Intervention Facilitators

The intervention will be delivered from the Krongold Clinic at Monash University, a university-based teaching and research clinic in Australia. Masters’ level psychology students will deliver the intervention under the supervision of qualified and experienced psychologists. Conduct of the trial will be led by the principal investigator and supported by a research team, all of whom will receive training in the requirements of the study protocol. Before training, all students were required to have successfully completed a 2-day workshop on assessing and responding to suicide and self-harm.

Experienced practitioners will deliver training specific to mi.spot over 2 days. The first day will focus on generic online counseling skills (in both group and individual counseling mode) and the second day will specifically examine the mi.spot intervention and how it should be delivered using simulated online sessions. Face-to-face training will be undertaken 2 weeks before starting the intervention. Fidelity checks for the 6 weekly sessions are built into the program at the facilitator level to ensure that all topics are covered. All features of the intervention are manualized, with guidelines provided for each function of the website.

### Safety

The safety protocol for the intervention consists of guidelines around privacy, online safety, and clinical safety. During the initial telephone call, during week 1, and as outlined under the online tab *things you need to know*, participants will be informed of and required to accept the terms of use for protecting their privacy and prohibited behaviors (ie, disrespectful, racist, or offensive comments or statements glorifying suicide or self-harm). The site will be checked, at a minimum twice a day, to ensure that there have been no rule violations (bullying, glorification of substance use, or self-harm). Participants will be informed that failure to comply may result in temporary or permanent withdrawal from the intervention.

In addition, every week, participants will be invited, via the site, to report any feelings of distress. If identified as being in distress, participants will be invited to an online one-to-one counseling session, with referrals provided if required. If a participant discloses statements indicating high distress (eg, suicidal ideation), the facilitator will conduct a telephone risk assessment and, where necessary, undertake 1 or more of the following procedures: (1) inform the supervisor, (2) inform the participant’s nominated emergency contact, and/or (3) liaise with suitable emergency services. Safety procedures are clearly outlined in the manual for facilitators to follow for any adverse event. Moreover, the 24/7 emergency numbers are visible under the tab *crisis contacts*.

Safety will be recorded in terms of the number of inappropriate posts (eg, bullying, the glorification of substance use, and self-harm), the tracking of participants’ mood over the 6 weeks, and participants’ retrospective reports of feeling safe/unsafe during interviews post the intervention.

### Study Population

The study includes 18- to 25-year-old (inclusive) Australians who identify as having a parent/caregiver with a mental illness and/or substance use concern (they do not have to be living with them). Potential participants will be contacted via telephone to ensure they are capable of providing informed consent and are currently not in distress or crisis (using self-report). They need to have access to a computer, mobile phone, or tablet and regular internet access. Those who reside outside Australia and cannot speak English are ineligible.

### Recruitment and Screening

Participants will volunteer following a response to social media, referral from health professionals, or word of mouth. Those interested in participating will be referred to the study webpage, which provides further information about the intervention. On the webpage participants are invited to confirm their age, contact details, parent’s mental health/substance use status, and emergency contact details. A link is provided to a consent form. Participants then complete all baseline questionnaires and are allocated to the intervention or wait-list control group. Once completed, participants will be contacted via telephone within 2 to 3 weeks by an intervention facilitator.

The telephone call with potential participants serves several purposes. It aims to ascertain participants’ expectations of the intervention and gives them an opportunity to ask any questions or voice any concerns they may have. The call helps to verify that the telephone number they provide is legitimate (important if the telephone number needs to be traced due to concerns regarding safety) and to confirm emergency contact details. During the call, the facilitator will gauge participants’ mental health status, their ability to provide informed consent, and whether the intervention is appropriate for them. They will do this by asking participants to self-report their current distress level (on a scale from 0 being no distress to 10 being high distress). The reference committee decided against using a validated mental health screen to exclude participants on the basis of their mental illness, given the high proportion of young adults who come from these families who have a mental health condition [22]. Nonetheless, it will be made clear to participants that the intervention is not a crisis service. If the facilitator assesses a participant as being in distress or in crisis, he/she will provide a referral to another appropriate service. Participants will also be told, during the call, which group they are in and whether they have any questions about this. Those in the control group will be notified that they are required to complete questionnaires in 7 to 8 weeks and also in 13 to 14 weeks.

### Allocation/Randomization

After completing baseline data collection, participants will be randomly allocated to 1 of the 2 study groups. Randomization will occur via a random number generator (using Statistical Package for Social Sciences), and participants will be allocated according to the timing (ie, order) of signing up for the study to intervention and control conditions. A permutation block of 70 will be used to ensure equivalence of intervention and control group allocations. The project manager will be responsible for the randomization, and the researchers will be blinded to the allocation of participants to the intervention and control conditions. The random number allocation procedure will occur before the commencement of the study. Participants will be informed about their allocation in the initial telephone call and follow-up email.

Those allocated to the intervention group will be provided with a link to the password-protected intervention. Participants in the control group will be given information about other local and national services they can access, including online and face-to-face services. They will be offered the intervention after those in the intervention group complete the postintervention questionnaires (approximately 12 weeks after randomization).

### Assessments

The data collection methods were developed and refined for acceptability from a previous pilot [21]. All participants will complete measures before randomization and at equivalent time frames post intervention and follow-up. Posttest completion will be immediately after the 6-week intervention (approximately 6-8 weeks after the completion of the prequestionnaire), and the follow-up questionnaire will be completed 6 weeks after the posttest. Participants will be sent online survey links and reminders using the REDCap database.

### Primary Outcome Measures

The primary outcome measures used in this study are as follows:

- The Mental Health Continuum short form is an internationally applied and thoroughly validated self-administered rating scale that contains items that measure 3 aspects of well-being: emotional, social, and psychological. Participants are asked the degree to which they have experienced emotional, social, and psychological well-being over the past month. The form includes a 6-point Likert scale from 0=never to 5 every day. Scores on this scale can range from 0 to 70, and higher scores indicate higher levels of well-being [23].
- The *Depression, Anxiety, and Stress Scale* is an internationally applied and thoroughly validated self-administered rating scale that measures levels of depression, anxiety, and stress. Participants are asked the degree to which they have experienced depression, anxiety, and stress over the past month. A 4-point Likert scale is used from 0 (did not apply to me at all) to 3 (applied to me very much or most of the time) within each of the 3 domains. Normal scores for depression, anxiety, and stress ranged from 0 to 4, 0 to 3, and 0 to 7, respectively, and scores above these ranges indicate mild to extremely severe levels in each domain [24,25].

### Secondary Outcome Measures

The secondary outcomes measures used in this study are as follows:

- The *Coping Orientation to Problems Experienced* inventory is an internationally applied measure that evaluates an individual’s levels of coping [26].
- The *General Help Seeking Questionnaire* is a measure that will be used to measure help-seeking behaviors [27].
- The *Social Connectedness Scale* will be used to measure an individual’s perceptions of social connectedness [28].
- The *Mental Health Literacy Scale* will be utilized to measure an individual’s level of psychoeducation [29].
- The *General Self-Efficacy Scale* will be employed to measure self-efficacy [30].
- The *Attribution of Responsibility for Parental Mental Illness Measure* was designed for the project to measure how responsible participants felt for their parents’ issue. The measure builds on attributional theory [31] and other research that has found that young people often blame themselves for their parent’s issue and consequently feel responsible for *fixing* it [32]. Examples of items were “I sometimes think my parent's illness is my fault.” Items are scored on a 7-point Likert scale ranging from 1 (strongly disagree) to 7 (strongly agree).

### Interviews: Determining Feasibility, Acceptability, and Safety

Individual interviews will be conducted with available (n=8) facilitators 1 to 2 weeks post the intervention to obtain their feedback on the training and the intervention in terms of feasibility, ease of use, practicality, and responsiveness. Individual interviews will also be conducted with 8 to 10 consenting mi.spot participants. Interview schedules for participants will be organized around safety as well as intervention acceptability, defined by the perceived benefit of the intervention and participants’ self-reported confidence in change [33]. If applicable, participants will be asked for their reasons for poor engagement or dropout.

### Usage

The total number and average length of log-ins will be recorded over 6 weeks. The number of attendees at each of the 6 weekly sessions, the number of participants who used the mi.thoughts.spot, and the number of participants who posted messages on the threads (including keeping track of repeat users) will be recorded. The number of facilitator-initiated and participant-initiated one-to-one sessions will also be recorded.

### Reimbursement

At the end of the 3 assessment periods, participants who complete all questionnaires will receive a AUD $50 (US $32) electronic gift voucher to use in selected stores (not for alcohol or tobacco). Intervention participants who engage in an interview will be paid with AUD $20 (US $12.85) voucher. Payment is provided in recognition of their time and to encourage completion/engagement.

### Participant Numbers

A total of 70 18- to 25-year-olds will be recruited. Participant numbers were initially determined by a power calculation indicating that a minimum of 44 participants, with Crit F=3.10 (using GPOWER 3.1, assuming 2 groups and 3 repetitions, a small effect size, an alpha of 5%, and power of 95%), will be required. However, based on previous dropout rates (the pilot study), it was considered that over the time frame of data collection (including the longer frame of the wait-list controls), there may be a dropout rate of up to 40%. As a consequence, recruitment numbers were increased to 70.

## Results

### Data Analysis

The impact of mi.spot on participants will be examined at pre, post, and follow-up time periods using analyses of variance on each of the measures outlined above. There will be a within-subjects factor (time) and a between-subjects factor (intervention/control). Data analysis will employ the intention-to-treat principle by including all participants in the analyses.

Interview data will be analyzed within a qualitative framework using interpretative phenomenological analysis (IPA). IPA is an approach that examines participants’ experiences and meanings of a phenomenon [34], and in this case, the facilitator’s and participants’ experiences of mi.spot. IPA also provides a structure for coding and categorization of data [34] and will be used to develop responses to questions regarding feasibility and acceptability. Before analysis, respondent validation will occur, a process that entails providing participants with a copy of their transcript and an invitation to delete any information they believe may be identifiable and/or modify existing or add any information. A second researcher will independently analyze one third of all transcripts. Rather than a numerical index of agreement, consensus will be reached by discussion and referring back to participants’ transcripts.

### Dissemination Strategy

The outcomes of the trial will be disseminated at conferences and in peer-reviewed journals. The general public, including young adults and other interested family members, mental health practitioners, and policy makers, will be notified of the study through public forums, government reports, policy statements, newsletters, and traditional and social media.

## Discussion

### Principal Findings

This paper describes the evaluation protocol for mi.spot, an online intervention for young adults aged 18-25 years whose parents have a mental illness and/or substance use concern. The intervention has a strong theoretical basis, which is lacking in most interventions in this area [35]. Given that 21% to 23% of all young people have a parent with a mental illness [2], such an initiative has the potential to make a substantial difference to the lives of many young people. The results of this study will add to the high-quality evidence base of electronic health interventions for this group of young people [36].

Notwithstanding its potential, the typical low rates of engagement in other online interventions for young people [37] are concerning. The flexible nature of the intervention in which participants can do some, all, or none of site features (and just *lurk*) may mitigate problems with engagement. Whether greater involvement equates to greater gains and relatedly determining how much engagement is sufficient to promote change are research questions that warrant further investigation.

As age-specific interventions increase young adults’ use of mental health services [38], interventions such as mi.spot may also promote the use of other, ongoing services for this group of young adults. Similarly, future investigations might investigate how online support could be integrated into face-to-face treatments and the types of referral pathways that are needed (both from and to mi.spot). Likewise, how an online intervention compares with similar face-to-face interventions [8] also remains to be investigated.

### Limitations

Participants report their own diagnoses and that of their parents, and these are not independently verified. The aims of the intervention are made clear from the outset, and thus, all participants (including those in the control group) will have some understanding of the nature of mi.spot that may encourage them to seek support elsewhere during the wait period and hence impact results [39]. Future considerations will need to investigate the cost effectiveness of the intervention and develop implementation guidelines to embed the intervention into routine care, which is important information for the long-term sustainable scale-up of effective public health interventions.

### Conclusions

The transition to adulthood can be a vulnerable period for young adults who have a parent with a mental illness or substance use concern. Given the issues related to stigma, access, and reach, online interventions hold great promise in engaging and intervening with this at-risk group. Support for the mi.spot trial will enhance the evidence base of a highly accessible intervention, which aims to prevent or reduce the adverse impact of young adults’ parents’ mental illness and/or substance use, for a large (approximately 21-23% of the population) high-risk group of young adults.

## Abbreviations

IPA: interpretative phenomenological analysis
mi.spot: mental illness; supportive, preventative, online, targeted
